# Supplementary material for: Development of Functional Msalais Wines Rich in Amadori Compounds by Yeast Fermentation
Source: Foods. 2025 Oct 11;14(20):3471. doi: 10.3390/foods14203471 (PMC12563688; doi:10.3390/foods14203471)
Supplement: Supplementary file 1 [file foods-14-03471-s001.zip › foods-3897638-supplementary.pdf]

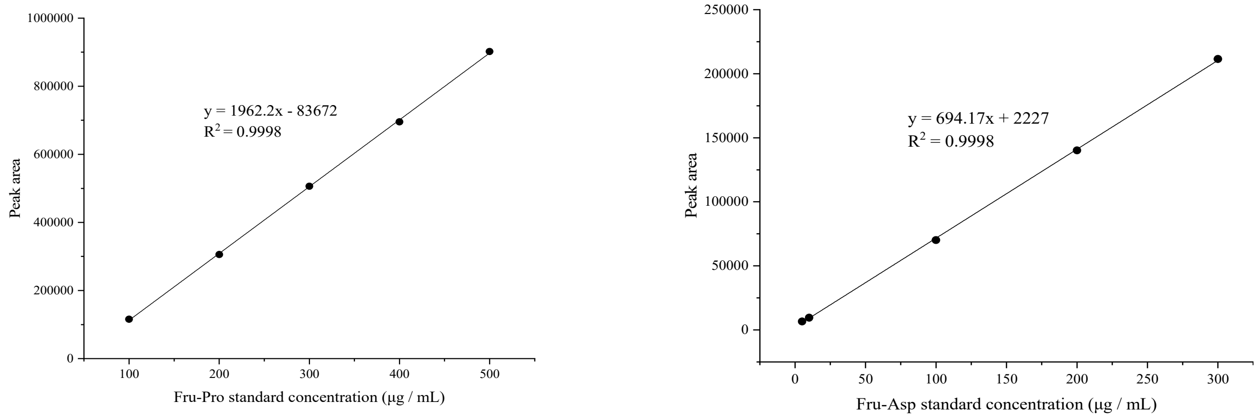

(**a**) Fru-Pro standard curve (**b**) Fru-Asp standard curve

Figure S1. Standard curves of two Amadori compounds.

Table S1. Accuracy test results of Fru-Pro and Fru-Asp.

Name	Known Concentration Number	Peak Area	Measured Value (g/L)	Average Measured Value (g/L)	Correlation Coefficient (R^2)	Standard Deviation (g/L)	Relative Standard Deviation (%)
Fru-Pro (0.25g/L)	1	405168	0.2492	0.2468	0.9998	0.0070	2.83
	2	384965	0.2389				
	3	411152	0.2522				
Fru-Asp (0.01g/L)	1	69154	0.0099	0.0110	0.9998	0.0011	9.49
	2	81432	0.0114				
	3	85319	0.0120				

Table S2. Precision test results of Fru-Pro and Fru-Asp

Name	Known Concentration Number	Peak Area	Measured Value (g/L)	Average Measured Value (g/L)	Correlation Coefficient (R^2)	Standard Deviation (g/L)	Relative Standard Deviation (%)
Fru-Pro (0.25g/L)	1	405168	0.2492	0.2397	0.9998	0.0110	4.59
	2	355947	0.2241				
	3	366948	0.2297				
	4	385264	0.239				
	5	410516	0.2519				
	6	395678	0.2443				
Fru-Asp (0.01g/L)	1	71125	0.0099	0.0101	0.9998	0.0009	8.72
	2	65415	0.0091				
	3	70185	0.0098				
	4	79263	0.0111				
	5	68894	0.0096				
	6	81021	0.0114				

Table S3. Repeatability test results of Fru-Pro and Fru-Asp

Name	Repeated Injection of the Same Sample Solution (Times)	Peak Area	Measured Value (g/L)	Average Measured Value (g/L)	Standard Deviation (g/L)	Relative Standard Deviation (%)
Fru-Pro	1	3899421	0.2030	0.2057	0.0055	2.67
	2	4121025	0.2143			
	3	3772005	0.1965			
	4	3921452	0.2041			
	5	3985241	0.2074			
	6	4012585	0.2088			
Fru-Asp	1	135777	0.0192	0.0186	0.0015	8.06
	2	145202	0.0206			
	3	121478	0.0172			
	4	115489	0.0163			
	5	128563	0.0182			
	6	140254	0.0199			

Table S4. Stability test results of Fru-Pro and Fru-Asp

Name	Placement Time (h)	Peak Area	Measured Value (g/L)	Average Measured Value (g/L)	Standard Deviation (g/L)	Relative Standard Deviation (%)
Fru-Pro	0	4213546	0.2190	0.2161	0.0070	3.25
	6	4225135	0.2196			
	12	3985621	0.2074			
	18	4002145	0.2082			
	24	4115836	0.2140			
	30	4337996	0.2253			
	36	4146713	0.2156			
Fru-Asp	0	130648	0.0185	0.0171	0.0015	8.71
	6	121582	0.0172			
	12	137254	0.0195			
	18	110565	0.0156			
	24	109521	0.0155			
	30	115754	0.0164			
	36	119659	0.0169			

Table S5. The recovery test results of Fru-Pro and Fru-Asp

	Quality in the Sample (mg)	The amount of Added Standard (mg)	The Quality Detected (mg)	Recovery Rate (%)	Average Value (%)	Standard Deviation (g/L)	Relative Standard Deviation (%)
Fru-Pro	0.2030	0.1250	0.3307	102.16	101.21	5.03	4.97
	0.2112	0.1250	0.3314	96.16			
	0.2157	0.1250	0.3389	98.56			
	0.2236	0.1250	0.3592	108.48			
	0.2045	0.1250	0.3251	96.48			
	0.2187	0.1250	0.3505	105.44			

Fru-Asp	0.0192	0.0500	0.0710	103.60	100.57	5.91	5.87
	0.0180	0.0500	0.0654	94.80			
	0.0197	0.0500	0.0735	107.60			
	0.0174	0.0500	0.0703	105.80			
	0.0165	0.0500	0.0655	98.00			
	0.0170	0.0500	0.0638	93.60			

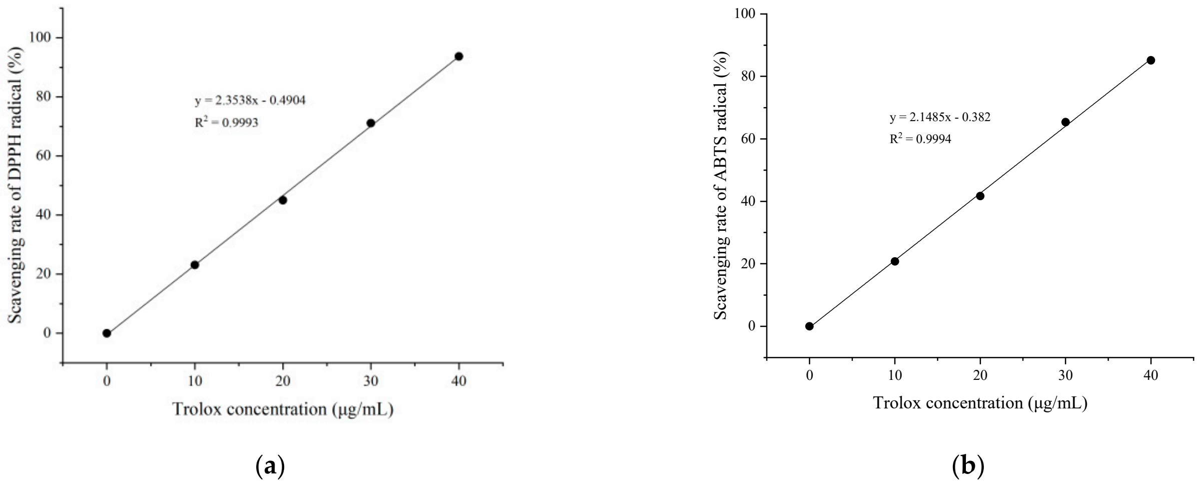

Figure S2. Trolox standard curve of DPPH free radical scavenging and ABTS free radical scavenging.

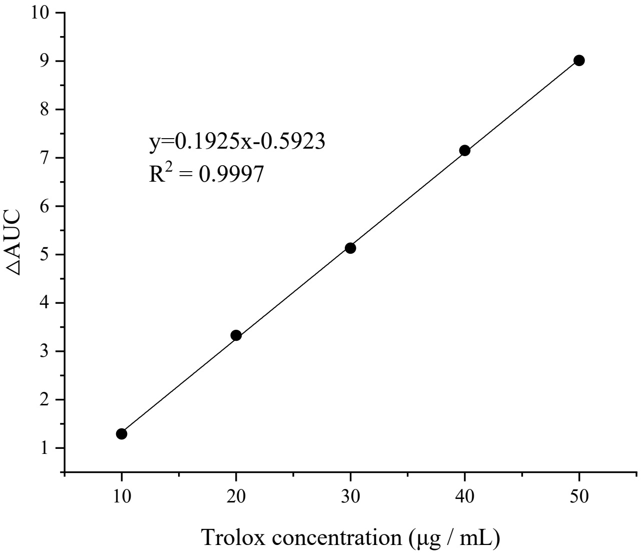

Figure S3. The Trolox standard curve of △AUC
